# Evidence from the Hamburg City Health Study – association between education and periodontitis

**DOI:** 10.1186/s12889-022-14096-7

**Published:** 2022-09-02

**Authors:** Carolin Walther, Kristin Spinler, Katrin Borof, Christopher Kofahl, Guido Heydecke, Udo Seedorf, Thomas Beikler, Claudia Terschüren, Andre Hajek, Ghazal Aarabi

**Affiliations:** 1 Department of Periodontics, Preventive and Restorative Dentistry, University Medical Center Hamburg-Eppendorf, Hamburg, Germany; 2 Institute of Medical Sociology, University Medical Center Hamburg-Eppendorf, Hamburg, Germany; 3 Department of Prosthetic Dentistry, Center for Dental and Oral Medicine, University Medical Center Hamburg-Eppendorf, Martinistraße 52, 20246 Hamburg, Germany; 4 Institute for Occupational and Maritime Medicine (ZfAM), University Medical Center Hamburg-Eppendorf (UKE), Hamburg, Germany; 5 Department of Health Economics and Health Services Research, Hamburg Center for Health Economics, University Medical Center Hamburg-Eppendorf, Hamburg, Germany

**Keywords:** Periodontitis, Oral health, Educational status, Risk factor, Cross -sectional study

## Abstract

**Objective:**

Large-scale population-based studies regarding the role of education in periodontitis are lacking. Thus, the aim of the current study was to analyze the potential association between education and periodontitis with state of the art measured clinical phenotypes within a large population-based sample from northern Germany.

**Material & methods:**

The Hamburg City Health Study (HCHS) is a population-based cohort study registered at ClinicalTrial.gov (NCT03934957). Oral health was assessed via plaque-index, probing depth, gingival recession and gingival bleeding. Periodontitis was classified according to Eke & Page. Education level was determined using the International Standard Classification of Education (ISCED-97) further categorized in “low, medium or high” education. Analyses for descriptive models were stratified by periodontitis severity. Ordinal logistic regression models were stepwise constructed to test for hypotheses.

**Results:**

Within the first cohort of 10,000 participants, we identified 1,453 with none/mild, 3,580 with moderate, and 1,176 with severe periodontitis. Ordinal regression analyses adjusted for co-variables (age, sex, smoking, diabetes, hypertension and migration) showed that the education level (low vs. high) was significantly associated with periodontitis (OR: 1.33, 95% CI: 1.18;1.47).

**Conclusion:**

In conclusion, the current study revealed a significant association between the education level and periodontitis after adjustments for a set of confounders. Further research is needed to develop strategies to overcome education related deficits in oral and periodontal health.

## Introduction

Periodontitis is a disease of the soft and hard tissue surrounding the tooth. Insufficient oral hygiene enables biofilm accumulation in deep periodontal pockets. This so-called “micro-ecosystem” can, under distinct environmental conditions, experience a shift towards the outgrowth of periodontal pathogen bacteria [1]. The clinical consequences are serious: destruction of the periodontium with tooth loss being the absolute endpoint of untreated disease manifestation, translocation of pathogenic bacteria into the bloodstream [2], secretion of pro-inflammatory cytokines that add to the overall sytemic inflammatory burden [3]. 42.2% of dentate US adults (between 35 -70 yrs old) suffer from periodontitis with 7.8% having a severe form [4]. Similar numbers have been observed in Germany: 8.2% of the younger population (35 – 44 yrs) and 19.8% of older individuals (65–74 yrs) are affected by severe periodontitis [5]. Disease onset and progression is highly dependent on endogenous and exogenous risk factors (e.g. diabetes mellitus, obesity, hypertension, smoking, oral hygiene and genetic deposition [6]), here listing only a small fraction of risks that the literature is reporting [7]. Currently, special attention is being paid to oral health literacy as a major risk for insufficient oral health. Oral health literacy (OHL) is defined as an individual’s capability to obtain, understand and process information in order to make appropriate and reasonable decisions regarding one´s own oral health. OHL is also crucial to navigate through the healthcare system for adequate support, treatment and care to achieve or maintain sufficient oral health [8]. Therefore, the level of education seems to be relevant for good oral health literacy. This notion is supported by a Brazilian cross-sectional study that reported a significant association between the number of years of education and higher OHL [9]. Moreover, several other studies showed a negative association between the degree of education and the ability to maintain periodontal health [10–13]. Data from the National Health and Examination Nutrition Surveys (NHANES) showed that higher levels of education – but not of income – were associated with greater odds of being periodontal healthy [14].

Therefore, it can be presumed that the level of education is a risk factor for periodontitis [15, 16] and that this relationship may be mediated by social inequalities and migration background. Since population-based data coming from large-scale population-based studies outside the United States are scarce, the aim of the current study was to determine the association between education and periodontitis in a large population-based sample from a European metropolitan area characterized by a prevention oriented health care system, statutory health insurance, and high utilization rates.

## Material & methods

### Subjects, study design and setting

Data was collected within the Hamburg City Health Study (HCHS), which is a prospective, long-term, ongoing population-based cohort study. This research platform was developed to expand knowledge about risk and prognostic factors of common chronic diseases. The involved random sample contains 10,000 participants of the general population of Hamburg, Germany, of which 6209 completed a full periodontal examination and were therefore included in the analysis. At sampling, participants were between 45 and 74 years of age. This sample took part in an extensive baseline assessment at one dedicated study center [17]. The institutional review board of the Medical Association of Hamburg approved the study protocol (PV5131). It was registered at ClinicalTrial.gov (NCT03934957). Participants were randomly selected via the residents' registration office and the response rate was 28%. This manuscript was prepared according to the STROBE guidelines [18].

### Assessment of education

Education level was classified according to the International Standard Classification of Education 2011 (ISCED 2011) and established by the United Nations Educational, Scientific and Cultural Organization (UNESCO) [19]. Eight levels of education are covered by this instrument: (0) Early childhood education, (1) Primary Education, (2) Lower secondary education, (3) Upper secondary education, (4) Post-secondary Non-Tertiary, (5) Short-cycle tertiary education, (6) Bachelor’s or equivalent level, (7) Master’s or equivalent level, (8) Doctoral or equivalent. For analyses, all participants were categorized in “low (0–2), medium (3–4) or high (5–8)” education.

### Assessment of dental variables

Certified study nurses performed the dental examination, which included: diagnosis of periodontitis with a standardized periodontal probe (CP-15 UNC SE, Hu-friedy, Chicago, USA) and a full mouth – six sites protocol, excluding the third molars. Periodontal parameters obtained were: 1) probing depths, 2) bleeding on probing (BOP), and 3) gingival recession. Oral hygiene was assessed via the oral plaque-index (PI). Additionally, the respective clinical attachment loss (CAL) was calculated for every tooth. The severity grading (none/mild, moderate, severe) of periodontitis was based on the classification of Eke & Page [20]:

1. Mild periodontitis: ≥ two interproximal sites with clinical attachment loss ≥ 3 mm, and ≥ two interproximal sites with probing depths ≥ 4 mm (not on the same tooth) or one site with probing depths ≥ 5 mm.
2. Moderate periodontitis: ≥ two interproximal sites with clinical attachment loss ≥ 4 mm (not on the same tooth), or ≥ two interproximal sites with probing depths ≥ 5 mm (not on the same tooth).
3. Severe periodontitis: ≥ two interproximal sites with clinical attachment loss ≥ 6 mm (not on the same tooth) and ≥ one interproximal site with probing depths ≥ 5 mm

Subsequently, the DMFT (D = decayed, M = missing, F = filled, T = teeth) was calculated. Participants requiring endocarditis prophylaxis were excluded from dental examination.

### Assessment of additional variables

The migration status was assessed with a self-administered questionnaire. Participants were asked about their own and their parents' place of birth. The answers were transferred into a binary variable (born in Germany/ born in a different country). Migration status was further classified into three categories: *immigrated* = participants were born outside of Germany and immigrated themselves; *migration background* = participants were born in Germany, but at least one parent was not born in Germany; *no migration background* = participants and both parents were born in Germany. Additionally, German language skills were conducted via self-assessment with a 5 point Likert-scale (very good – very poor). Additional variables were assessed at baseline: age (years) and sex (male/female) as well as cardiovascular risk factors: BMI (kg/m^2^), smoking yes/no, diabetes (positive self-disclosure, taking medication of the A10 group (Anatomical Therapeutic Chemical Classification System (ATC-Code)), fasting glucose (> 126 mg/dl), not fasting glucose (> 200 mg/dl)), coronary artery disease (CAD), and hypertension. Blood samples were obtained for biomarker analysis (high-sensitive C-reactive protein (hs-CRP) and Interleukin 6 (IL-6)) and stored at -80 °C at the HCHS Biobank. Further, plasma samples were analyzed using established enzyme-linked immunosorbent assays (ELISA).

### Statistical analyses

In descriptive analyses, continuous variables are presented with their medians and interquartile ranges (IQR). Similarly, absolute numbers (n) and percentages (%) are presented for categorical variables. Descriptive analyses were presented for all variables stratified by the grading of periodontitis (none/mild, moderate and severe) and differences within groups were tested using the chi-squared test or Kruskal–Wallis test. Ordinal logistic regression models were conducted with the outcome variable “periodontitis severity” and the exposure variable “education”. Models with adjustments for relevant confounders (age, sex, history of ever smoking, diabetes, hypertension, migration status, and education) were applied based on prior research and clinical rationale. A *p*-value of < 0.05 was considered statistically significant. Statistical analyses were performed using R software, version 4.1.0.

## Results

### Descriptive statistics

1453 participants with none/mild, 3580 with moderate, and 1176 with severe periodontitis were identified within the 10,000 cohort. Compared to participants with none/mild periodontitis, participants with severe periodontitis were older (66 years), more frequently men (60.9%), had more cardiovascular relevant comorbidities (BMI = 26.4, smoking = 25.1%, diabetes = 11.3%, hypertension = 72.5%), and more often a diagnosed cardiovascular disease = 9.3%. This trend was also apparent for IL-6 (participants with severe periodontitis = 1.77; participants with none/mild periodontitis = 1.45) and CRP (severe = 0.13; none/mild = 0.10). Dental variables, especially the plaque-index (severe = 22; none/mild = 0), differed between the two groups, with the severe group having the highest scores for all variables (Table 1).

**Table 1:** Baseline characteristics stratified by periodontitis severity

Periodontitis:	None/mild	Moderate	Severe	*p*-value for Trend
**n**	1453	3580	1176	
	*n (%); median [IQR]*	*n (%); median [IQR]*	*n (%); median [IQR]*	
***DEMOGRAPHICS***				
**Female sex**	878 (60.4)	1814 (50.7)	460 (39.1)	< 0.001
**Age**	59 [52,66]	63 [55,69]	66 [59,71]	< 0.001
**Education**				< 0.001
**Low**	410 (29.5)	1197 (35.0)	461 (41.6)	
**Medium**	295 (21.2)	640 (18.7)	187 (16.9)	
**High**	686 (49.3)	1579 (46.2)	461 (41.6)	
**Not born in Germany**	176 (12.7)	485 (14.2)	161 (14.6)	0.311
**Years living in Germany**	37.00 [26.00,49.00]	39.00 [27.00,53.50]	38.00 [25.00,49.00]	0.339
**Migration**				0.050
**Immigrated**	176 (16.0)	485 (19.1)	161 (20.6)	
**Migration background**	83 (7.5)	161 (6.3)	44 (5.6)	
**No migration background**	842 (76.5)	1894 (74.6)	575 (73.7)	
**German language skills**				0.603
**Very good**	45 (42.9)	112 (44.1)	34 (37.0)	
**Good**	39 (37.1)	87 (34.3)	32 (34.8)	
**Moderate**	19 (18.1)	50 (19.7)	24 (26.1)	
**Poor**	2 (1.9)	1 (0.4)	1 (1.1)	
**Very poor**	0 (0.0)	4 (1.6)	1 (1.1)	
***LABORATORIES***				
**IL-6**	1.45 [1.01,2.04]	1.55 [1.15,2.16]	1.77 [1.33,2.63]	< 0.001
**Hs-CRP**	0.10 [0.06,0.23]	0.11 [0.06,0.25]	0.13 [0.07,0.30]	< 0.001
**Total energy (kcal/day)**	1989.2 [1568.7,2543.3]	2047.6 [1628.7,2593.8]	2068.9 [1639.3,2602.3]	0.057
***DENTAL VARIABLES***				
**DMFT Index**	17.00 [14.00,21.00]	19.00 [16.00,23.00]	21.00 [17.00,24.25]	< 0.001
**BOP Index**	2.08 [0.00,7.14]	8.33 [2.17,19.23]	21.05 [9.26,41.67]	< 0.001
**Plaque-index**	0.00 [0.00,10.71]	8.93 [0.00,27.78]	22.00 [5.77,54.76]	< 0.001

*Abbreviations*: *BMI* Body Mass Index, *BOP* Bleeding on probing, *CAD* Cardiovascular diseases, *DMFT* Decayed, Missing, Filled, Teeth, *IQR* Interquartile range, *IL-6* Interleukin 6, *Hs-CRP* High sensitive – c-reactive protein, *p*-value for Trend: differences within groups were tested using the chi-squared test or Kruskal–Wallis test

Within participants with severe periodontitis, 26.1% presented moderate German language skills, whereas among participants with none/mild periodontitis, 18.1% presented moderate German language skills (Table 1). Furthermore, 47.1% of participants with low education answered the question “have you ever had periodontal therapy?” with yes; those with high education were 42.3%. Only 64.8% of participants with low education answered the question “Do you have your teeth professionally cleaned at least once a year?” with yes, while 75.9% participants with high education answered positively on this question. 14.9% of participants with low education visited the dentist predominantly when they experienced pain or discomfort, in the group with high education it was 13.5%.

### Regression analysis

Ordinal logistic regression analyses revealed a significant association between education level and periodontitis, when comparing low to high education level (OR = 1.41, *p* < 0.001). After stepwise adjusting for co-variables (age, sex, smoking, diabetes, hypertension and migration), the probability of participants with low education level having periodontitis was still significantly higher (OR = 1.33, *p* < 0.001) (Table 2).

**Table 2:** Ordinal logistic regression analysis of association between periodontitis and education level

Variable	Units	OR [95%CI]	*p*-value
**Model 1**			
**Education**	**Low**	1.41 [1.29;1.52]	< 0.001
	**Medium**	0.96 [0.82;1.09]	0.537
	**High**	Ref	
**Model 2**			
**Education**	**Low**	1.33 [1.18;1.47]	< 0.001
	**Medium**	1.08 [0.91;1.25]	0.381
	**High**	Ref	
**Age**		1.05 [1.04;1.06]	< 0.001
**Sex**	**Male**	Ref	
	**Female**	0.58 [0.45;0.71]	< 0.001
**Smoking**	**No**	Ref	
	**Yes**	1.85 [1.68;2.02]	0.001
**Diabetes**	**No**	Ref	
	**Yes**	1.03 [0.78;1.28]	0.803
**Hypertension**	**No**	Ref	
	**Yes**	1.21 [1.07;1.34]	0.007
**Migration status**	**No migration background**	Ref	
	**Immigrated**	1.14 [0.98;1.30]	0.115
	**Migration background**	0.89 [0.64;1.14]	0.365

Model 1: unadjusted. Model 2: fully adjusted

## Discussion

In the current study, participants with severe periodontitis were more frequently older men, with more cardiovascular comorbidities and weaker oral hygiene. Ordinal logistic regression analyses revealed a significant association between education level (low vs. high) and periodontitis, even after adjusting for co-variables (age, sex, smoking, diabetes, hypertension and migration).

Data (*n* = 13,665) from the National Health and Nutrition Examination Survey III (NHANES III) also revealed that individuals with low SES scores and with low education were more likely to have periodontitis. In this study, low education increased the risk for periodontitis by three times (OR = 3.12, 95% CI = 2.40, 4.06) compared to the higher educated [14]. However, when comparing NHANES data with our findings, we have to consider two aspects: NHANES participants were younger compared to our sample (NHANES: mean, SE 40.1 ± 0.37); and the survey period lasted from 1988–94. Therefore, a meaningful comparison is problematic due to a lack of contemporary results.

The health care system in Germany is organized differently from that in the Unites States. Germany has a much more prevention oriented health care system and the statutory health insurance pays for basic care (e.g. regular check-ups, acute pain therapy, amalgam fillings, extractions). Low education is strongly associated with a low socioeconomic status (SES) [21]. Patients with lower SES usually present lower oral-health literacy [22], meaning how they understand, regular attend and utilize health information and prevention programs. Consequently, those disadvantaged members of society still experience barriers to attend regular dental check-ups/treatment: (1) Many dental services (e.g. prosthetic dentistry) must be paid out of the pocket by patients [23]. Patients with lower income usually cannot afford this extra payment. (2) Lower physician-population ratio in socially deprived districts [24, 25]. In this context, lower SES is often documented in migration groups [26] and therefore we chose to include migration background as a potential confounder. However, in the sub-group of participants with severe periodontitis we only had small samples sizes of immigrated participants (*n* = 161) and participants with migration background (*n* = 44), and the effect of a migration background as a potential confounder might not be significant because of a lack of power.

Evidence regarding the oral health status in citizens with lower education is highly necessary, because oral health does affect general health [27]. Via translocation of pathogen bacteria or increasing pro-inflammatory cytokines, periodontitis is known to be associated with cardiovascular diseases (atherosclerosis, arterial hypertension, atrial fibrillation), diabetes mellitus type 2, rheumatoid arthritis and psoriasis [28]. Further research and political decision-making need to focus on this accumulation of risk factors in order to promote equality of oral health opportunities [29].

### Limitations

The current study has some limitations. Because all participants had to read, understand and answer the self-questionnaire regarding education level, we could not include participants with relatively poor or no German language skills. To enable comparability of our results with other epidemiological studies, severity grading (none/mild, moderate, severe) of periodontitis was based on the classification for epidemiological studies [30] and not based on the 2017 developed case definition for periodontitis [31]. Furthermore, this is a cross-sectional study design. It is therefore not possible to draw causal conclusions.

## Conclusion

In conclusion, the current study revealed a significant association between the education level and periodontitis after adjustments for a set of confounders. Further research is needed to develop strategies to overcome education related deficits in oral and periodontal health.

## Data Availability

The datasets generated and/or analysed during the current study are not publicly available due to legal restrictions, but are available from the corresponding author on reasonable request.
